# Advances in Alzheimer's Disease: From Bench to Bedside

**DOI:** 10.1155/2015/202676

**Published:** 2015-02-19

**Authors:** Teng Jiang, Raymond Chuen-Chung Chang, Hanna Rosenmann, Jin-Tai Yu

**Affiliations:** ^1^Department of Neurology, Qingdao Municipal Hospital, Nanjing Medical University, Nanjing 210006, China; ^2^Laboratory of Neurodegenerative Diseases, Department of Anatomy, LKS Faculty of Medicine, The University of Hong Kong, Pokfulam, Hong Kong; ^3^Department of Neurology, The Agnes Ginges Center for Human Neurogenetics, Hadassah Hebrew University Medical Center, Ein Karem, 91120 Jerusalem, Israel; ^4^Department of Neurology, Qingdao Municipal Hospital, School of Medicine, Qingdao University, Qingdao 266000, China; ^5^Department of Neurology, Memory and Aging Center, University of California, San Francisco, San Francisco, CA 94143, USA

In 1906, Alois Alzheimer first documented the case of Auguste Deter, a patient with a combination of cognitive deficits, psychiatric symptoms, and microscopic brain lesions. This histopathological and clinical constellation was then designated by Emil Kraepelin as Alzheimer's disease (AD) [1]. Nowadays, AD became the most common progressive neurodegenerative disease and the most common form of dementia among the elderly [2, 3]. In the clinic, the late-onset AD (LOAD) accounts for 95% of all AD cases and is currently considered as a genetic complex disorder that is probably caused by a combination of multiple risk alleles and environmental factors [4]. To date,* APOE* is the only unequivocally established susceptibility gene for LOAD [5]. However, it has been estimated that the variations of* APOE* account for less than 50% of LOAD risk, suggesting that there are additional genetic risk factors which remain to be uncovered. Recent advances in genetic approaches led to the identification of numerous risk genes for AD, which has greatly extended our knowledge on the genetic components of this disease [6]. In this special issue, the paper entitled “Clinical Genetics of Alzheimer's Disease” by Z. Zou et al. outlined these novel susceptibility genes, such as* CLU*,* CR1*,* CD33*,* PICALM*,* BIN1*,* TREM2*, and* PLD3*. More importantly, they summarized the recent evidence regarding the functions of these genes as well as their association with phenotypes and pathogenesis of AD. This allowed readers to get a good understanding on the research advances in the genetic basis and pathological mechanisms of this devastating disease.

Regarding the neuropathology, AD is mainly characterized by the formation of extracellular neuritic plaques containing the amyloid-*β* peptide (A*β*) and intraneuronal accumulation of neurofibrillary tangles constituted by hyperphosphorylated tau protein. In 2002, John Hardy proposed “amyloid hypothesis,” which emphasized A*β* accumulation as the initial pathological events in the progression of AD [7]. Therefore, therapeutic strategies against A*β*, especially A*β*-induced neurotoxicity, have attracted a lot of attention in recent years. In the current issue, by employing primary neuron culture, C.-F. Lau et al. provided the first evidence that testosterone could protect against A*β*-induced synaptic dysfunction and degeneration in “Protective Effects of Testosterone on Presynaptic Terminals against Oligomeric *β*-Amyloid Peptide in Primary Culture of Hippocampal Neurons.” These exciting findings emphasized testosterone as a potential endogenous target of drug development, which might open up a new avenue for the prevention and treatment of AD. Additionally, in this issue, another original paper entitled “Inhibitory Effects of Edaravone in *β*-Amyloid-Induced Neurotoxicity in Rats” by F. He et al. provided preclinical evidence concerning the beneficial effects of edaravone, a free radical scavenger mainly used for stoke treatment, in an AD model induced by A*β* injection. For the first time, they showed that edaravone could attenuate A*β*-induced increase of voltage-gated calcium channel currents and cholinergic neurons losses, which subsequently improved learning and memory performance. Their interesting finding implied that edaravone might have a practical clinical use for AD prevention and treatment, emphasizing the notion that many therapeutic agents possessed pleiotropic actions in addition to their main applications.

In addition to its own neurotoxicity, A*β* as the central pathological factor also initiates a series of secondary events in AD progression, such as neuroinflammation. Activation of microglia, the main immune cell in the brain, is considered as a central event in A*β*-induced neuroinflammation. Actually, several lines of evidence suggested a “double-edged sword” function of microglia during the progression of AD [8]. On one hand, long-term A*β* stimulation results in the dysfunction of microglia in the brain, which is characterized by the overproduction of proinflammatory cytokines, subsequently leading to the bystander neuronal and synaptic damage. On the other hand, activated microglia participates in the phagocytosis of A*β* through its phagocytic activity and thus prevents the deposition of A*β* and the formation of amyloid plaques. In this special issue, a review article entitled “Microglia in Alzheimer's Disease” by Y. Li et al. summarized the recent advances concerning microglia during AD progression. Meanwhile, they also introduced the recent basic and clinic efforts regarding how to prevent and treat this disease via precise modulation of microglial functions. Their contributions will greatly help the readers to get a better understanding on the role of microglia and neuroinflammation in the mechanisms and therapeutics of AD.

In summary, the articles in this issue cover the recent progress in the genetic basis and molecular mechanisms underlying AD pathogenesis, accompanied with the development of diagnostic approaches and therapeutic strategies for this disease. We hope that the reader will extend their knowledge about the basic and clinical aspects of AD through this collection of articles.

*Teng Jiang* *Raymond Chuen-Chung Chang* *Hanna Rosenmann* *Jin-Tai Yu*
